# Commentary: Effects of acid-sensing ion channel-1A (ASIC1A) on cocaine-induced synaptic adaptations

**DOI:** 10.3389/fphys.2023.1295561

**Published:** 2023-12-04

**Authors:** Abbas Bader, Abdul Yousaf, Xiang-Ping Chu

**Affiliations:** Department of Biomedical Sciences, University of Missouri-Kansas City School of Medicine, Kansas City, MO, United States

**Keywords:** acid-sensing ion channels, cocaine addiction, synaptic adaptation, neuronal signaling, striatum, dopamine

## Introduction

Among Americans, cocaine continues to be reported as the most commonly used illicit stimulant (Mustaquim et al., 2021). From 2018 to 2019, the estimated prevalence of cocaine usage within the past year among U.S. adults was 2.14% (Mustaquim et al., 2021). There remain no approved pharmacotherapies for stimulant use disorders, including cocaine use, perpetuating the persistence of a public health crisis (Sofuoglu and Sewell, 2009; Chu, 2017). However, acid-sensing ion channels (ASICs) provide an area of increasing novel interest related to drug-addiction behaviors. ASICs are a group of proton-sensing Na^+^ channels that are formed through the trimeric combination of various homo/heteromeric subunits into a functional channel (Waldmann et al., 1997; Wemmie et al., 2013; Kreple et al., 2014b; Storozhuk et al., 2021). ASICs function as homeostatic pH sensors that are transiently activated during physiological conditions of extracellular acidosis (Waldmann et al., 1997; Chu and Xiong, 2012; Wemmie et al., 2013). In particular, ASIC1A subunits are highly expressed in the nucleus accumbens core (NAc core), a region of the mesolimbic pathway widely implicated in drug-addiction behaviors and reward circuits (Adinoff, 2004; Kreple et al., 2014a). Previous studies have suggested that ASIC1A inhibits neuroplasticity in neural reward pathways of drug-addiction behaviors, thus potentially promoting addiction-related neuroprotection (Kreple et al., 2014b; Gupta et al., 2023). ASIC1A likely plays a vital role in maintaining synaptic maturity and stability in the NAc (Yu et al., 2018). Inversely, ASIC1A disruption in mice produces synaptic rearrangements in the NAc comparable to cocaine-withdrawn mice. ASIC1A null mice exhibit increased cocaine-conditioned place preference (CCP), dendritic spine density, rectification index (RI), and AMPAR/NMDAR ratio (Kreple et al., 2014a; Gupta et al., 2023). ASIC1A disruption is analogous to a state of increased cocaine-evoked plasticity, reasoning that ASIC1A has clear therapeutic potential that can be leveraged to reduce addiction vulnerability (Kreple et al., 2014b; Heusser and Pless, 2021).

## Time-dependent effects of cocaine on synaptic physiology following ASIC1A disruption

A recent study reported in *Frontiers in Physiology* conducted by Dr. Wemmie’s Laboratory provides further insights into the role of ASIC1A in synaptic neuroadaptations (Gupta et al., 2023). The study introduced ASIC1A disruption and examined the temporal effects of single cocaine dose exposure (10 mg/kg) on the synaptic architecture of C57BL/6J mice models. The study demonstrates that ASIC currents, ASIC-mediated EPSCs, and ASIC1A protein bands were all absent in the ASIC1A^−/−^ mice. Intraperitoneal injections of either cocaine (10 mg/kg) or saline (0.9%) were administered, followed by electrophysiological recordings performed on coronal NAc brain slices at post-injection intervals of 6 h, 24 h, and 7 days. Additionally, AMPAR/NMDAR ratio and AMPAR rectification in NAc neurons was recorded at post-injection intervals of 6 h, 15 h, 24 h, 4 days, and 7 days. The findings revealed that at 6 h post-injection, the ASIC1A^−/−^ cocaine group had an unchanged AMPAR/NMDAR ratio compared to the ASIC1A^−/−^ saline control group and remained elevated compared to the wildtype ASIC1A^+/+^ control groups. However, the AMPAR/NMDAR ratio of the ASIC1A^−/−^ cocaine group significantly decreased 15 h post-injection until 4 days, returning to baseline levels at 7 days. The study suggests that the increased recruitment of GluA2-lacking calcium-permeable AMPA receptors (CP-AMPAR) contributes to the elevated AMPAR/NMDAR ratio in ASIC1A null mice. The increased synaptic recruitment of CP-AMPAR causes structural changes in AMPAR’s tetrameric subunit configuration. NASPM was utilized as a selective antagonist of CP-AMPAR and higher levels of NASPM sensitivity were found in ASIC1A null mice, demonstrating the increased recruitment of CP-AMPAR. Next, the temporal effects of a single cocaine injection on the dendritic spine density of NAc core medium spiny neurons (MSN) was examined. The ASIC1A^−/−^ saline group showed an increase in total dendritic spine density compared to wild-type. The ASIC1A^−/−^ cocaine group showed a significant decrease in total dendritic spine density including mushroom, thin, and stubby morphological classes when compared to the ASIC1A^−/−^ saline group. Moreover, Gupta et al. examined if ASIC1A disruption is cell autonomous or if it induces downstream secondary effects. ASIC1A disruption was introduced in the NAcc by injecting an eGFP control vector expressing Cre recombinase into mice. Results showed that ASIC currents were terminated in MSNs transduced with eGFP^+^ Cre, but remained in non-transduced MSNs or those expressing only the eGFP control vector. The Cre-transduced neurons exhibited significantly higher AMPAR/NMDAR ratios than those transduced with the control vector, supporting the notion that ASIC1A disruption has cell-autonomous effects. Furthermore, this study also found increased changes in synaptic plasticity among D1R MSNs lacking ASIC1A. This was done by comparing ASIC1A^−/−^ crossed mice differing in D1R expression. This study found that the AMPAR/NMDAR ratio was greater among D1R^+^ mice compared to D1R^-^ mice. Lastly, protein synthesis inhibitor anisomycin was found to help reverse increased AMPAR/NMDAR ratio and RI in ASIC1A^−/−^ mice which is comparable to aniscomycin’s effect on cocaine-withdrawn mice.

## Discussion

A study conducted by *Gupta et al.* has identified multiple novel characteristics of ASIC1A^−/−^ NAc MSNs in relation to cocaine withdrawal and acute cocaine injection (Gupta et al., 2023). These novel characteristics include studying the temporal effects of single cocaine administration, determining whether ASIC1A disruption leads to cell autonomous effects, measuring an increased specificity of dopamine 1 receptor (D1R) expressing MSNs, and sensitivity towards anisomycin (Gupta et al., 2023). ASIC1A disruption and associated changes in synaptic plasticity, including changes in AMPAR/NMDAR ratio, were found to be cell-autonomous, indicating that ASIC1A plays a key role within intracellular signaling for cocaine withdrawal resistance instead of providing secondary extracellular effects. MSNs within the NAc are well connected and play an essential role in the integration of the striatal complex and receive mesencephalic, cortical, and thalamic afferents helping regulate many forms of behavior including addiction (Diana, 2011; Spiga et al., 2014). Thus, possible targeted therapies for ASIC1A could avoid unexpected side effects from connecting neural circuits and pathways due to its cell-autonomy. Moreover, ASIC1A disruption within the Nac disproportionately affected D1R^+^ MSN more which are known to be excitatory G-protein coupled receptors implicated in the progression of CCP and sensitization in contrast to D2R^+^ MSNs (Hikida et al., 2010; Lobo et al., 2010; Ferguson et al., 2011; Smith et al., 2013). ASIC1A’s seemingly protective effects against cocaine withdrawal appears to be correlated with D1R^+^ MSN pointing to a possible inhibitory relationship between both of them. Further studies testing the physiological role of ASIC1A within D1R^+^ could help give more insight into the mechanisms of drug addiction and potential therapeutic targets. One target explored was an extracellular pH-buffering enzyme called carbonic anhydrase 4 (CA4); inhibition of this enzyme could lead to a decrease in pH, causing increased activation of ASIC1A. *Gupta et al.* found NAc CA4 inhibition prevented AMPAR/NMDAR ratio increase following cocaine injection and prevented cocaine seeking behavior following 4 weeks withdrawal (Gupta et al., 2022). However, confounding evidence has been presented by *Gutman et al.* and *Jiang et al. Gutman et al.* demonstrated that ASIC1A overexpression in the NAc core caused potentiation of cocaine seeking behavior after 3 weeks of withdrawal (Gutman et al., 2020). Likewise, *Jiang et al.* found decreased locomotor sensitization among ASIC1A^−/−^ mice compared to WT mice in the final injection following a 2 weeks withdrawal from 5 days daily cocaine administration exemplifying the need for further ASIC1A research identifying physiological nuances with ASIC1A overexpression (Jiang et al., 2013). ASIC1A disruption has also been studied in relation to morphine as well and shows an increase on morphine-conditioned place preference (Kreple et al., 2014a). Overall, ASIC1A may be involved broadly in drug related addiction, underscoring its importance for future research and study.
